# European adults’ physical activity socio-demographic correlates: a cross-sectional study from the European Social Survey

**DOI:** 10.7717/peerj.2066

**Published:** 2016-06-02

**Authors:** Adilson Marques, João Martins, Miguel Peralta, Ricardo Catunda, Luís Saboga Nunes

**Affiliations:** 1 Centro Interdisciplinar de Estudo da Performance Humana, Faculdade de Motricidade Humana, Universidade de Lisboa, Lisboa, Portugal; 2 Centro de Investigação em Saúde Pública, Escola Nacional de Saúde Pública, Universidade Nova de Lisboa, Lisboa, Portugal; 3 Laboratório de Pedagogia, Faculdade de Motricidade Humana, Universidade de Lisboa, Cruz Quebrada, Portugal; 4 UIDEF, Instituto de Educação, Universidade de Lisboa, Lisboa, Portugal; 5 Faculdade de Motricidade Humana, Universidade de Lisboa, Cruz Quebrada, Portugal; 6 Centro de Ciências da Saúde, Universidade Estadual do Ceará, Fortaleza, Brazil

**Keywords:** Health promotion, Leisure time physical activity, Lifestyles, Physical inactivity

## Abstract

**Background.** From a public health perspective, the study of socio-demographic factors related to physical activity is important in order to identify subgroups for intervention programs.

**Objective.** This study aimed to identify the prevalence of, and the socio-demographic correlates related to, the achievement of recommended physical activity levels.

**Methods.** Using data from the European Social Survey round 6, physical activity and socio-demographic characteristics were collected, in 2012, from 39,278 European adults (18,272 men, 21,006 women), aged 18–65 years, from 28 countries. The question of meeting physical activity guidelines was assessed using World Health Organization criteria.

**Results.** A total of 64.50% (63.36% men, 66.49% women) attained physical activity recommended levels. The likelihood of attaining physical activity recommendations was higher in the 55–64 years age group (men: OR = 1.22, *p* < 0.05; women: OR = 1.66, *p* < 0.001), among those who had secondary education (men: OR = 1.28, *p* < 0.01; women: OR = 1.26, *p* < 0.05), among those who lived in rural areas (men: OR = 1.20, *p* < 0.001; women: OR = 1.10, *p* < 0.05), and among those who had three or more people living at home (men: OR = 1.40, *p* < 0.001; women: OR = 1.43, *p* < 0.001). On the other hand, attaining physical activity recommendations was negatively associated with being unemployed (men: OR = 0.70, *p* < 0.001; women: OR = 0.87, *p* < 0.05), being a student (OR = 0.56, *p* < 0.001; women: OR = 0.64, *p* < 0.01), being a retired person (men: OR = 0.86, *p* < 0.05) and with having a higher household income (OR = 0.80, *p* < 0.001; women: OR = 0.81, *p* < 0.01).

**Conclusions.** This research helped clarify that, as the promotion of physical activity is critical to sustain health and prevent disease, socio-demographic factors are important to consider when planning the increase of physical activity.

## Introduction

The health benefits of physical activity (PA) are well established (Hardman & Stensel, 2009). Nonetheless, approximately one third of adults worldwide do not exercise enough to benefit their health (European Commission, 2014; Hallal et al., 2012). Studies based on self-reported PA in Australia (Rosenberg et al., 2010), Canada (Bryan & Katzmarzyk, 2009), the United States of America (Tucker, Welk & Beyler, 2011), and in Europe (Gerovasili et al., 2015; Marques et al., 2015) have shown that about 40% of the adults are considered not physically active.

Due to the evidence of PA health benefits, and the high prevalence of inactivity, national and international agencies have produced consensus statements on the central role of promoting PA in the adult population as part of an effort to reduce premature mortality and morbidity associated with chronic diseases (European Union, 2008; USDHHS, 2008; WHO, 2010). The European Union, the United States Department of Health and Human Services, and the Word Health Organization are among the many agencies which have recommended that all healthy adults should participate in at least 30 min of moderate-intensity aerobic activity, at least five days per week, to promote health (European Union, 2008; USDHHS, 2008; WHO, 2010). These recommendations emphasize the benefits of moderate intensity, and provide an innovative aspect related to the accumulation of PA throughout the day.

For effective public health surveillance and interventions, it is important to determine not only the proportion of people who participate in PA, but also to understand the factors related to the practice of PA of those who meet the PA recommended level. A better understanding of the contributing factors related to PA participation is critical to designing policies and effective interventions because it allows researchers to pay attention to modifying factors. To help identify subgroups for intervention programs, one must study the prevalence of, and socio-demographic factors related to, PA as it pertains to: sex, age, education level, living location, partnership status, the presence or absence of children in the home, household number, citizenship, and household income (Bauman et al., 2012; Belanger, Townsend & Foster, 2011; Kamphuis et al., 2009; Marques et al., 2014). Exploring the socio-demographic correlates of PA could help to understand the participation factors. Knowing the factors associated with PA would enable to identify disadvantaged groups and tailor interventions that would target populations with the identified characteristics. Therefore, this study aimed to identify the socio-demographic correlates related with PA recommended levels.

## Methods

### Study design and participants

This is a cross-sectional multi-country study based on data from the European Social Survey round 6, 2012, comprising 28 European countries and Israel (Albania, Belgium, Bulgaria, Cyprus, Czech Republic, Denmark, Estonia, Finland, France, Germany, Hungary, Iceland, Ireland, Israel, Italy, Kosovo, Lithuania, Netherlands, Norway, Poland, Portugal, Russian Federation, Slovakia, Slovenia, Spain, Sweden, Switzerland, Ukraine, United Kingdom). The European Social Survey is an academically driven cross-national survey that has been conducted every two years across Europe since 2001. The survey measures the attitudes, beliefs and behaviour of European people.

Probability sampling from all residents aged 15 years and older was applied in all countries, comprising 54,673 participants. Sample were representative of all persons aged 15 and over resident within private households in each country. Individuals were selected by strict random probability methods. In all countries there was a minimum effective achieved sample size of 1,500 or 800 in countries with populations of less than 2 million. For the present study, only adults were selected since the PA recommendations for youth and older people are different from adults. Thus, participants younger than 18 years of age (*n* = 2,000) and older than 65 years of age (*n* = 10,779) were excluded from the sample. Since Israel is not a European country, its citizens were excluded (*n* = 2,508). Finally, those who did not report at least four socio-demographic characteristics were also excluded (*n* = 108). These restrictions resulted in a final sample size of 39,278 participants (18,272 men, 21,006 women) with mean age 41.85 ± 13.62 (men, 41.58 ± 13.49; women, 42.09 ± 13.25).

### Measures

#### Physical activity

Information on PA was assessed with a single item asking, “On how many of the last seven days did you walk quickly, do sports, or other PA for 30 min or longer?” Although PA was assessed with a single item, there is evidence that in studies where PA is not the primary focus, and more detailed measures are not feasible, a single question is an acceptable alternative (Wanner et al., 2014). Using the World Health Organization (WHO, 2010) criteria, participants were classified as having attained the recommended level of PA (≥30 min of at least moderate PA on five or more occasions per week), or not having attained the PA recommended levels (<30 min of at least moderate PA on five or more occasions per week).

#### Socio-demographic characteristics

Participants reported their sex and age. Using reported ages, participants were categorized into five age groups (18–24, 25–34, 35–44, 45–64). The European Social Survey data provides two variables of education attainment: the recoded variable that focuses on levels of education achieved and years of full time education. For the analysis, the level of education achieved was used, as the population might cluster according to it (Carlson et al., 2010; Marques et al., 2014). Participants were classified as: primary (less than high school), secondary (high school education), and tertiary (superior education). Participants were asked to report what they had been doing for the last seven days. Response options were: paid work (employed), studying (education), unemployed actively looking for a job, unemployed but not actively looking for a job, retired, military service, and others. Both unemployed categories were classified into a single category: unemployed. Those who were doing military service were considered employed. To determine the living place, participants were asked to report whether they lived in a big city, a suburb/outskirts of a big city, a town or small city, a country village, or a home in the countryside. Those who responded that they lived in a big city, or the suburbs/outskirts of big city, were grouped into a new category: urban areas. Those who indicated that they lived in a country village, or in a home in the countryside, were grouped into a category called, “rural areas.” Respondents were asked to describe whether they live with or without a husband/wife/partner, and the legal situation. Response options were dichotomized into live with or without a partner. Participants answered if they lived with or without children at home, and then the number of people living regularly as a member of the household. In each country, participants were asked whether they were national citizens or immigrants. Household income was determined based on decile calculated in each country separately. Using this data, 1st to 3rd decile, 4th to 7th decile, and 8th to 10th were organized to create three groups.

### Procedures

The European Social Survey is an open database for free access. We obtained access through the following link: http://www.europeansocialsurvey.org/data/download.html?r=6.

The European Social Survey uses a multi-stage probability cluster sampling design to provide national representative samples. According to national options, participants were sampled by means of postal code address files, population registers, social security register data, or telephone books. In the sampling procedure, statistical precision was kept the same for all countries, notwithstanding the difference in method used for a specific country. In each country, information was collected using a questionnaire (European Social Survey, 2012) completed through an hour-long face-to-face interview that included questions on the use of medicine, immigration, citizenship, socio-demographic and socioeconomic issues, health perception, and PA. Appropriate ethical consent, from ethical committees were gained in each participating country.

### Statistical analysis

Descriptive statistics were calculated for all variables (means, standard deviation, and percentages). Mann–Whitney test and Chi-square test were used to compare men and women according to socio-demographic characteristics and PA. ANOVA, followed by Tukey’s HSD test; Student *t*-test were performed to assess socio-demographic variables for the number of times participants engaged in PA in the last 7 days. Bivariate relationships between PA (not attaining the PA recommended level vs. attaining the PA recommended level) and socio-demographic variables were tested by Chi-square test and Fisher’s exact test. To analyse the effects that socio-demographic variables had on attaining PA recommended levels, a binary logistic regression analysis was conducted. The binary logistic regression was adjusted for country and age. All analyses were stratified by sex, and statistical analysis was performed using IBM SPSS Statistics 22. The significance level was set at *p* < 0.05.

**Table 1:** Participants’ socio-demographic characteristics.

	Total (*n* = 39,278)	Men (*n* = 18,272)	Women (*n* = 21,006)	*p*
	*n* (%)	*n* (%)	*n* (%)	
Age^a^				0.011
18–24	5,445 (13.86)	2,667 (14.60)	2,778 (13.22)	
25–34	7,293 (18.57)	3,415 (18.69)	3,879 (18.46)	
35–44	8,631 (21.97)	3,934 (21.53)	4,697 (22.36)	
45–54	9,321 (23.73)	4,306 (23.57)	5,015 (23.87)	
55–64	8,588 (21.86)	3,950 (21.62)	4,638 (22.08)	
Education level^b^				<0.001
Primary	2,193 (5.61)	940 (5.17)	1,253 (5.99)	
Secondary	27,467 (70.28)	13,279 (73.10)	14,188 (67.83)	
Tertiary	9,425 (24.11)	3,948 (21.73)	5,477 (26.18)	
Occupation^b^				<0.001
Employed	24,253 (69.55)	12,371 (81.32)	11,882 (67.81)	
Unemployed	4,233 (12.11)	2,074 (11.96)	2,159 (12.32)	
Students	3,552 (10.19)	1,702 (9.81)	1,852 (10.57)	
Retired	2,829 (8.11)	1,199 (6.91)	1,631 (9.31)	
Living place^b^				0.007
Urban area	12,967 (33.09)	5,969 (32.74)	6,999 (33.40)	
Town or small city	11,885 (30.33)	5,447 (29.87)	6,439 (30.73)	
Rural areas	14,331 (36.57)	6,817 (37.39)	7,515 (35.87)	
Partnership status^b^				0.340
Live without partner	13,754 (35.15)	6,357 (34.91)	7,397 (35.37)	
Live with partner	25,372 (64.85)	11,855 (65.09)	13,518 (64.63)	
Children living at home^b^				<0.001
No	19,643 (49.99)	10,094 (55.25)	9,549 (45.46)	
Yes	19,633 (51.01)	8,177 (44.75)	11,456 (54.54)	
Members of household^b^				<0.001
1 person	4,135 (10.53)	2,135 (11.68)	2,000 (9.52)	
2 people	10,205 (25.98)	4,607 (25.22)	5,598 (26.65)	
3–4 people	18,661 (47.51)	8,791 (48.11)	9,870 (46.99)	
≥5 people	6,277 (15.98)	2,739 (14.99)	3,538 (16.84)	
Citizenship status^b^				0.090
National	37,482 (95.49)	17,399 (95.30)	20,082 (95.66)	
Immigrant	1,770 (4.51)	858 (4.70)	912 (4.34)	
Household income^b^				<0.001
1st to 3rd decile	8,613 (27.19)	3,757 (25.24)	4,855 (28.92)	
4th to 7th decile	13,215 (41.72)	6,139 (41.23)	7,077 (42.16)	
8th to 10th decile	9,847 (31.09)	4,993 (33.53)	4,854 (28.92)	
PA in the last 7 days (≥30 min/day)^a^				<0.001
None	2,586 (6.58)	1,152 (6.31)	1,434 (6.83)	
1 day	1,674 (4.26)	865 (4.74)	809 (3.85)	
2 days	2,867 (7.30)	1,402 (7.67)	1,464 (6.97)	
3 days	3,686 (9.38)	1,777 (9.72)	1,906 (9.07)	
4 days	3,134 (7.98)	1,497 (8.19)	1,636 (7.79)	
5 days	4,512 (11.48)	2,298 (12.58)	2,211 (10.53)	
6 days	3,089 (7.86)	1,530 (8.37)	1,559 (7.42)	
7 days	17,743 (45.16)	7,750 (42.42)	9,988 (47.55)	
PA recommendations^b^				<0.001
Not attaining PA recommended level	13,942 (35.50)	6,694 (36.64)	7,248 (34.51)	
Attaining PA recommended level	25,336 (64.50)	11,578 (63.36)	13,758 (65.49)	

**Notes.**

PA physical activity

Attaining physical activity recommended level means ≥30 min of at least moderate physical activity, five or more times per week. Not attaining physical activity recommended levels means <30 min of at least moderate physical activity, five or more times per week.

a Tested by Mann–Whitney test.

b Tested by Chi-square.

**Table 2:** Estimated prevalence of European people’s physical activity in the last seven days by socio-demographic characteristics.

	Men (*n* = 18, 272)		Women (*n* = 21, 006)	
	Number of times of PA in the last seven days (≥30 min/day) (95% CI)	*p*	Number of times of PA in the last seven days (≥30 min/day) (95% CI)	*p*
Age^a^		0.005		<0.001
18–24	4.81 (4.72–4.89)		4.58 (4.50–4.67)	
25–34	4.86 (4.79–4.94)		4.99 (4.92–5.06)	
35–44	4.92 (4.84–4.99)		5.12 (5.05–5.19)	
45–54	4.98 (4.91–5.05)		5.15 (5.08–5.21)	
55–64	4.98 (4.91–5.06)		5.26 (5.19–5.32)	
Education level^a^		<0.001	<0.001
Primary	4.51 (4.34–4.68)		4.77 (4.62–4.91)	
Secondary	5.06 (5.02–5.09)		5.17 (5.13–5.21)	
Tertiary	4.55 (4.48–4.63)		4.85 (4.79–4.91)	
Occupation^a^		<0.001		<0.001
Employed	5.02 (4.98–5.06)		5.10 (5.06–5.14)	
Unemployed	4.80 (4.69–4.90)		5.05 (4.95–5.15)	
Students	4.62 (4.51–4.72)		4.44 (4.34–4.54)	
Retired	4.98 (4.84–5.12)		5.39 (5.27–5.50)	
Living place^a^		<0.001		<0.001
Urban area	4.84 (4.79–4.90)		5.00 (4.94–5.05)	
Town or small city	4.76 (4.70–4.82)		4.93 (4.87–4.98)	
Rural areas	5.11 (5.06–5.17)		5.24 (5.18–5.29)	
Partnership status^b^		<0.001		<0.001
Live without partner	4.82 (4.76–4.87)		4.83 (4.78–4.89)	
Live with partner	4.98 (4.94–5.02)		5.18 (5.14–5.22)	
Children living at home^b^		0.001		<0.001
No	4.87 (4.82–4.91)		4.90 (4.85–4.94)	
Yes	4.99 (4.94–5.04)		5.20 (5.16–5.24)	
Members of household^a^		<0.001		<0.001
1 person	4.75 (4.65–4.85)		4.85 (4.74–4.95)	
2 people	4.87 (4.80–4.94)		5.08 (5.02–5.14)	
3–4 people	4.96 (4.92–5.01)		5.03 (4.98–5.08)	
≥5 people	5.00 (4.92–5.09)		5.24 (5.17–5.32)	
Citizenship status^b^		0.700		0.825
National	4.92 (4.89–4.96)		5.06 (5.03–5.09)	
Immigrant	4.89 (4.74–5.04)		5.05 (4.89–5.20)	
Household income^a^		<0.001		0.005
1st to 3rd decile	4.87 (4.79–4.94)		5.09 (5.02–5.16)	
4th to 7th decile	4.98 (4.92–5.04)		5.08 (5.03–5.14)	
8th to 10th decile	4.79 (4.72–4.85)		4.96 (4.89–5.02)	

**Notes.**

PA physical activity

a Tested by ANOVA, followed by Tukey’s HSD test.

b Tested by *t*-test for independent samples.

## Results

The general samples’ characteristics are presented in Table 1. On average, men had participated 4.92 times per week in PA in the last seven days, while women had participated 5.06 per week (*t*(39,276) = − 6.036, *p* < 0.001). Significantly less men (63.36%) than women (65.49%) attained the PA recommended levels (*χ*^2^(1) = 19.379, *p* < 0.001).

**Table 3:** Binary logistic regression predicting the attainment of the physical activity recommended level by European people.

	Attaining the PA recommended level OR (95% CI)	
	Men	Women
Age		
18–24	1.00 ref.	1.00 ref.
25–34	0.98 (0.83–1.14)	1.14 (0.97–1.34)
35–44	1.03 (0.87–1.21)	1.26 (1.06–1.50)^**^
45–54	1.09 (0.92–1.29)	1.41 (1.19–1.67)^***^
55–64	1.22 (1.02–1.45)^*^	1.66 (1.39–1.99)^***^
Education level		
Primary	1.00 ref.	1.00 ref.
Secondary	1.28 (1.08–1.51)^**^	1.26 (1.04–1.52)^*^
Tertiary	0.76 (0.63–0.91)^**^	0.90 (0.74–1.10)
Occupation		
Employed	1.00 ref	1.00 ref
Unemployed	0.70 (0.62–0.79)^***^	0.87 (0.78–0.98)^*^
Students	0.56 (0.47–0.66)^***^	0.64 (0.55–0.75)^**^
Retired	0.86 (0.73–1.00)^*^	1.08 (0.92–1.25)
Living place		
Urban area	1.00 ref.	1.00 ref.
Town or small city	0.93 (0.85–1.02)	0.93 (0.85–1.01)
Rural areas	1.20 (1.10–1.30)^***^	1.11 (1.02–1.22)^*^
Partnership status		
Live without partner	1.00 ref.	1.00 ref.
Live with partner	1.12 (0.99-1.27)	1.08 (0.98–1.18)
Children living at home		
No	1.00 ref.	1.00 ref.
Yes	0.76 (0.66–0.87)	1.12 (1.00–1.25)
Members of household		
1 person	1.00 ref.	1.00 ref.
2 people	0.97 (0.83–1.13)	1.00 (0.87–1.15)
3–4 people	1.28 (1.10–1.50)^**^	1.05 (0.89–1.24)
≥5 people	1.40 (1.17–1.67)^***^	1.43 (1.18–1.73)^***^
Citizenship status		
National	1.00 ref.	1.00 ref.
Immigrant	1.00 (0.85–1.02)	1.05 (0.88–1.25)
Household income	1.00 ref.	1.00 ref.
1st to 3rd decile	1.00 ref.	1.00 ref.
4th to 7th decile	0.93 (0.84–1.02)	0.94 (0.86–1.04)
8th to 10th decile	0.80 (0.72–0.89)^***^	0.81 (0.72–0.90)^***^

**Notes.**

PA physical activity OR odds ratio CI confidence interval

Attaining the physical activity recommended level means ≥30 min of at least moderate physical activity, five or more times per week. Analyses were adjusted for country and age.

* *p* < 0.05.

** *p* < 0.01.

*** *p* < 0.001.

The average time that men (*F*(4, 18,266) = 79.139, *p* = 0.005) and women (*F*(4, 21, 001) = 889.932, *p* < 0.001) participated in PA for at least 30 min increased significantly with age (Table 2). Those with secondary education engaged more frequently in PA than those with primary and tertiary education (men: *F*(2, 18, 163) = 934.613, *p* < 0.001; women: (*F*(2, 20,915) = 506.854, *p* < 0.001). Employed men (*F*(3, 17, 344) = 19.696, *p* < 0.001) and retired women (*F*(3, 17, 522) = 58.473, *p* < 0.001) practiced PA more often than others with a different occupation. Men and women from rural areas were more physically active (men: *F*(2, 18, 228) = 433.770, *p* < 0.001; women: *F*(2, 20, 948) = 376.616, *p* < 0.001). Similarly, those who lived with a partner (men: *t*(18,210) = 4.510, *p* < 0.001; women: *t*(20,913) = 10.410, *p* < 0.001), had children (men: *t*(18,269) = 3.452, *p* = 0.001; women: *t*(21,004) = 9.387, *p* < 0.001), and lived with more people at home (men: *F*(3, 18, 267) = 108.164, *p* < 0.001; women: *F*(3, 21,002) = 221.176, *p* < 0.001), engaged significantly more times in PA than individuals who lived without any partner, had no children and had less members in the household. Men with an income between decile 4th and 7th (*F*(2, 14, 886) = 104.335, *p* < 0.001), and women with 1st to 3rd and 4th to 7th decile (*F*(2, 16,783) = 55.731, *p* = 0.005), were more active than those from other income levels.

Table 3 presents the results of the multivariate binary logistic regression. For men, being in the age group of 55–64 years was positively related to attaining PA recommended levels (OR = 1.22, 95% CI [1.02–1.45], *p* < 0.05), compared to the younger age group. Attaining PA recommendations was also positively associated with: secondary education (OR = 1.28, 95% CI [1.08–1.51], *p* < 0.01), living in rural areas (OR = 1.20, 95% CI [1.10–1.30], *p* < 0.001), and having three or more people living at home (OR = 1.28, 95% CI [1.10–1.50], *p* < 0.01; and OR = 1.40, 95% CI [1.17–1.67], *p* < 0.001). On the other hand, attaining the recommended levels of PA was negatively associated with: tertiary education (OR = 0.76, 95% CI [0.63–0.91], *p* < 0.01), being unemployed (OR = 0.70, 95% CI [0.62–0.79], *p* < 0.001), being a student (OR = 0.56, 95% CI [0.47–0.66], *p* < 0.001), and being a retired person (OR = 0.86, 95% CI [0.73–1.00], *p* < 0.05), when compared with being employed; and with having a higher household income (OR = 0.80, 95% CI [0.72–0.89], *p* < 0.001).

For women, older ages were more likely to attain the recommended levels of PA, and the age group of 55–64 years represented the strongest association (OR = 1.66, 95% CI [1.39–1.99], *p* < 0.001). Furthermore, attaining the PA recommendation was more likely among those with secondary education (OR = 1.26, 95% CI [1.04–1.52], *p* < 0.05), who lived in rural areas (OR = 1.10, 95% CI [1.02–1.20], *p* < 0.05), and who had five or more people living at home (OR = 1.43, 95% CI [1.18–1.73], *p* < 0.001). Conversely, women who were unemployed (OR = 0.87, 95% CI [0.78–0.98], *p* < 0.05), students (OR = 0.64, 95% CI [0.55–0.75], *p* < 0.01), and had the highest household income (OR = 0.81, 95% CI [0.72–0.90], *p* < 0.01) had a lower likelihood of attaining PA recommended levels.

## Discussion

The present study examined the associations of socio-demographic factors with engagement in the recommended PA level among European adults. The results showed that 64.5% attained the PA recommended levels. Age, education level, occupation, living place, number of household members, and household income are factors related to PA participation, and are related to attaining the PA recommended levels among European adults.

European women were significantly more active than men, and were also more likely to meet the PA guidelines. This finding is different from other studies, which showed that men were more likely to engage in PA that met the guidelines (Hallal et al., 2012; Murtagh et al., 2015a.; Tucker, Welk & Beyler, 2011). Although these results are not in line with most studies, it cannot be said that the outcome is entirely different from the literature. Studies among adults from Portugal, The Netherlands, Luxembourg, Lorraine (France) and Wallonia (Belgium) showed that women slightly surpassed men in time spent in PA during leisure time (Alkerwi et al., 2015; Marques et al., 2014; Mesters, Wahl & Keulen, 2014). The increased activity among women could be due to extra available time caused by a variable workload at home, and caring for children. Another reason could be that men achieve their PA levels by playing sports, but with increasing age these activities become harder to continue.

In contrast to other studies (Bauman et al., 2012; Carlson et al., 2010; Murtagh et al., 2015a.), this study showed that PA participation in at least 30 min per day increased as age increased, as did the proportion of people attaining the PA recommended level. These findings are particularly interesting because the aging of the population has social and economic implications (including an increase in age-related diseases), and PA contributes to health promotion and disease prevention (Woodcock et al., 2011). Perhaps the increase of PA with age is related to the fact that older people more often visit family doctors, who are likely to recommend PA as part of the patient’s everyday work (Bull et al., 1997; Suija et al., 2010). For this population, PA has much to offer in terms of personal and public health, as it helps to prevent some important age-related diseases, while enhancing functional capacities, which leads to a better quality of life as well as an increased capacity for independent living (Murtagh et al., 2015b). Notwithstanding, it should be noted that this study measured PA participation in at least 30 min per day and not total PA participation, which could explain the differences found with previous studies.

Those with secondary education were more likely to be physically active. Previous investigations showed that participants who achieved a higher educational level showed a lower prevalence of a sedentary lifestyle (Bauman et al., 2012). However, there are also studies that do not observe a relationship between these variables (Marques et al., 2014; Shibata et al., 2009). The correlation between education level and PA is not entirely understood beyond the fact that it is reported as a correlate of activity, but not determinant (Bauman et al., 2012). In this particular study, the results should be interpreted carefully because of the wide cultural variance among countries. Nonetheless, for European people in general, one can speculate that people with a higher education level generally have high control, high daily demands, and long work hours. These realities might reduce their available time for PA.

Employed and retired individuals were more active than students and the unemployed. The PA levels of the employed could be due to active commuting or, in some cases, the demands of the workplace. It is plausible that most students were young adults and were studying at university. Regular PA during this stage of transition into adulthood serves as an important foundation for adult life patterns (Telama et al., 2014). Further, this group may be important since those who attend university may play an important role in establishing social and cultural norms as they move into roles as decision-makers and opinion leaders within the population (Leslie et al., 1999). For unemployed people, results confirmed what was observed among adults from the United States of America (Van Domelen et al., 2011). The unemployed do not accumulate any occupational PA, or any activity associated with daily commuting. As a result, leisure time is the primary opportunity for PA. Unfortunately, unemployment is associated with depression (Khlat, Sermet & Le Pape, 2004), which is related with less PA during leisure time (Song et al., 2012). Furthermore, many times unemployed have limited financial resources to join sport and fitness clubs, which could difficult participation in PA. This is a group at risk, and strategies to minimize the effect of unemployment on PA participation have to be developed.

People from rural areas were more active than those from other areas. This could be due to the fact that in rural areas more people, mainly men, work in the primary and secondary sectors of the economy, thereby increasing PA both in the workplace and in the household (Fan, Wen & Kowaleski-Jones, 2014). This is particularly important because the urban population in 2014 accounted for 54% of the total global population, and it is estimated that, by 2017, a majority of people will be living in urban areas. This data suggests that the prevalence of PA may decrease as a result of growing urbanization.

Due to the complexity of addressing social structural determinants of health, PA research focuses mainly on individual-level factors. However, there is an increased emphasis on the role of social factors as modifiable determinants of PA (McNeill, Kreuter & Subramanian, 2006). Interpersonal relationships may affect PA by providing social support and establishing social norms that compel or facilitate health-promoting behaviours (Silva, Azevedo & Goncalves, 2013). The results of this study suggest that a higher number of individuals in a household is correlated with attaining the PA recommended levels. Previously it was observed that, as a category, women living alone was negatively associated with PA, unlike men living alone (Murtagh et al., 2015a.). Perhaps in some countries women without partners were particularly disadvantaged in terms of their living standards, which may have an impact on access to PA participation.

Higher household income was negatively associated with attaining the PA recommended levels. This study’s findings do not support the idea that people from higher income or socioeconomic status are more physically active (Dias da Costa et al., 2005; Wilson et al., 2004). So far there is no consensual evidence that socioeconomic status explains people’s PA behaviours. Nevertheless, neighbourhood aesthetics, street connectivity, safety from crime, and proximity to parks are all associated with recreational walking and PA (Kamphuis et al., 2009; Sugiyama et al., 2014). Environmental factors may explain the variance in PA among socioeconomic status categories, observed in some studies, since access to attractive, safe, green space and resources for structured PA may be limited in deprived areas. In cases where people from a lower household income are less physically active than those from a higher household income, interventions to reduce differences in the availability of recreational PA among adults would be effective if they focused on neighbourhood perceptions as well as individual cognition (Kamphuis et al., 2009).

The current investigation had some strengths and limitations that have to be addressed. A major strength of the study is that the European Social Survey database includes a large and representative sample size of various European countries, as well as several socio-demographic characteristics to characterize the study sample. PA was self-reported rather than objectively measured, which could be subject to bias. People’s self-reported PA may be overestimated because of social desirability (Sallis & Saelens, 2000). However, there is evidence that social desirability accounts for only a small variance in PA (Motl, McAuley & Distefano, 2005), and self-reported is a reliable method for epidemiologic studies (Craig et al., 2003), even when using a single item (Wanner et al., 2014). The analysis was cross-sectional, thereby making it impossible to determine cause and effect. Furthermore, there was no information about the participants’ weight status. This would be of importance since weight status is related with PA (Carlson et al., 2010).

## Additional Information and Declarations

### Data Availability

The following information was supplied regarding data availability: The data used in this research is publicly available from The European Social Survey: http://www.europeansocialsurvey.org/data/download.html?r=6.
